# How undergraduate art students evaluate ChatGPT-generated art interpretations: perceived quality, bias-related judgment, and rating structure

**DOI:** 10.3389/fpsyg.2026.1828685

**Published:** 2026-08-07

**Authors:** Hui-Chao Tian, Xiao-Hui Li

**Affiliations:** School of Fine Arts and Design, Xuchang University, Xuchang, Henan, China

**Keywords:** AI-generated interpretation, art education, bias-related judgment, generative AI, perceived quality

## Abstract

**Introduction:**

Generative artificial intelligence increasingly produces explanatory texts for artworks, but how students evaluate these texts remains insufficiently understood. This study examined undergraduate art students' evaluations of ChatGPT-generated art interpretations when the AI source was explicitly disclosed.

**Methods:**

A total of 134 students evaluated Chinese-language interpretations of 10 artworks associated with United States art history. For each artwork, students reported their familiarity and rated school or period identification, formal analysis detail, content or theme interpretation, inferential rigor, cultural or stylistic conjecture, learning inspiration, and bias-related perceived quality.

**Results:**

Overall evaluations were moderately positive but varied across rating items. School or period identification and formal analysis detail received relatively high ratings, whereas the bias-related item, D7, received the lowest mean and greatest dispersion. Because all items were positively keyed, lower D7 scores indicated greater perceived bias-related problems. Internal consistency, correlation, and principal component analyses showed substantial overlap among D1–D6, indicating a broad textual-quality impression, whereas D7 was less strongly integrated with this response pattern. Artwork familiarity, prior AI-based artwork-analysis experience, and art history or visual culture coursework were associated with more favorable ratings. General AI-use frequency showed no significant adjusted association.

**Discussion:**

Under disclosed-source conditions, the findings show how students organize perceived-quality judgments of AI-generated art interpretations. Such interpretations may support observation, discussion, and evidence-verification activities in art education.

## 1 Introduction

Generative AI now participates in art education as a producer of explanatory language as well as images. ChatGPT can organize an artwork interpretation around style, form, theme, and context, giving students terminology and an initial route into analysis (Kasneci et al., 2023; OpenAI et al., 2023). Its linguistic fluency can also obscure weak evidence, unsupported contextual claims, or cultural overgeneralization. Evaluating an AI-generated interpretation therefore involves both its immediate usefulness and the grounds on which its claims are made.

Art interpretation contains tasks with different evidential demands. Formal features such as composition, color, brushwork, and spatial organization can be checked against the visible work. School attribution draws on recognizable stylistic and historical categories. Thematic, cultural, and intentional claims require contextual knowledge and a warranted connection between evidence and interpretation. Leder et al.'s (2004) account of aesthetic processing organizes this progression from perceptual analysis and classification to cognitive mastering and evaluation. This progression provides a conceptual basis for comparing students' judgments across interpretive tasks.

Generative outputs introduce two practical concerns for these tasks. First, plausible language may contain factual errors or hallucinated support (Ji et al., 2023; Kasneci et al., 2023). Cecilio-Fernandes and Sandars (2025) describe this mismatch as a “hallucination of learning,” in which fluent assistance can create an impression of understanding without secure knowledge. Second, learners may rely on outputs more confidently when they possess enough prior knowledge to verify them. In a classroom study of ChatGPT-5, students who encountered hallucinations reported restricting use to areas where they could check the answer, a strategy described as epistemic safeguarding (Sivenas, 2025). Perceived helpfulness is therefore educationally relevant when considered alongside students' capacity to examine evidence and contextual claims.

Source disclosure supplies the context in which these judgments are made. Studies comparing AI and human labels show that attribution can alter evaluations of artworks (Ragot et al., 2020; Neef et al., 2025). Here, students knew that each interpretation was generated by ChatGPT before rating it. The findings consequently characterize evaluative responses under a disclosed AI source; estimating a labeling effect would require human-labeled or unlabeled comparison conditions.

The analysis asks how undergraduate art students rate seven aspects of ChatGPT-generated interpretations. These include school or period identification, formal analysis detail, thematic interpretation, inferential rigor, contextual conjecture, learning inspiration, and bias-related perceived quality. All seven items are positively keyed. A high D7 score means that the text is perceived as avoiding cultural simplification, stereotyping, and overgeneralization; a low score means that more such problems are perceived. Repeated ratings of 10 artworks serve three analytical purposes. They support item-level comparisons, describe the response structure, and examine associations with familiarity, disciplinary background, AI-art-analysis experience, AI training, self-rated competence, and general AI-use frequency.

## 2 Theoretical framework

### 2.1 Aesthetic processing and the formation of explanations

Aesthetic processing and art criticism pedagogy both describe art understanding as an integration of perception, classification, interpretation, and judgment. Viewers identify formal cues, connect them with prior experience, and locate the artwork within stylistic or historical categories. Cultural knowledge and historical context then contribute to thematic interpretation and evaluation (Leder et al.'s, 2004; Leder and Nadal, 2014). Art criticism similarly distinguishes description, formal analysis, interpretation, and judgment while recognizing their interaction in practice (Feldman, 1994; Barrett, 2002). Together, these accounts position art interpretation as a sequence of related tasks whose evidential requirements become more demanding as claims move from visible form to contextual meaning.

Visible features and recognizable stylistic categories provide relatively direct reference points for evaluating an interpretation. Theme, cultural context, inferential rigor, and bias-related quality require broader knowledge and closer attention to uncertainty (Radford et al., 2021; Li et al., 2023). AI-generated prose can present these different claims with similar linguistic confidence, potentially encouraging a general impression of quality even when their evidential support varies (Ji et al., 2023). The seven items therefore sample linked aspects of students' evaluation, from art-historical positioning and formal description to contextual inference, learning inspiration, and bias-related quality. Tables 1, 2 provide the corresponding conceptual and operational definitions.

**Table 1:** Mapping between aesthetic-processing-related tasks and the seven rating items.

Aesthetic-processing-related task	Corresponding rating item	What this study measures	Interpretive boundary
Explicit classification/art-historical positioning	D1 School or period identification (Gombrich, 2004)	Students' perception of whether the AI's school or period identification is reasonable	Student-rated classification quality; objective accuracy requires expert verification
Perceptual analysis/description of formal cues	D2 Formal analysis detail (Barnet, 2014)	Students' perception of whether the AI describes visible features such as composition, color, line, and spatial organization	Perceived formal-description quality
Content or theme interpretation/thematic understanding	D3 Content or theme interpretation (Panofsky, 1993)	Students' perception of whether the AI's content or theme interpretation shows depth	Perceived interpretive depth
Inferential rigor	D4 Inferential rigor (Toulmin, 2003)	Students' perception of whether the AI's reasoning is clear and evidence-based	Perceived argumentative quality
Contextual integration and uncertain inference	D5 Cultural or stylistic conjecture (Panofsky, 1993)	Students' perception of whether the AI's cultural or stylistic links are appropriate and constrained	Perceived contextual-inference quality
Learning inspiration	D6 Learning inspiration (Efland, 2002)	Students' perception of whether the AI text supports further understanding or reflection	Perceived learning inspiration; learning outcomes require direct performance measures
AI bias-related quality judgment	D7 Bias-related perceived quality (Bender et al., 2021)	Students' perception that the AI text avoids cultural simplification, stereotyping, or overgeneralization	Positively keyed perceived quality; bias-detection competence requires a separate measure

**Table 2:** Operational definitions of the seven rating items.

Rating item	What is rated	High-score characteristics	Low-score characteristics
D1 School or period identification	Whether the AI's identification of style, school, or historical period is perceived as reasonable	The interpretation is perceived as placing the work reasonably within a stylistic or historical lineage	The classification is perceived as vague, weakly supported, or unreasonable
D2 Formal analysis detail	Whether the AI's description of composition, color, line, and other formal elements is perceived as specific and relevant	The interpretation is perceived as describing salient visible formal features in a specific way	The description is perceived as generic or as omitting key visible elements
D3 Content or theme interpretation	Whether the AI's interpretation of subject matter, narrative, or symbolism is perceived as meaningful	The interpretation is perceived as engaging with narrative, symbolism, or thematic meaning	The interpretation is perceived as remaining at the level of surface description
D4 Inferential rigor	Whether the reasoning chain from formal cues to content or theme interpretation is perceived as clear and warranted	Claims are perceived as logically connected to evidence, with a clear relation between observed cues and interpretive conclusions	Reasoning is perceived as jumpy, weakly justified, or insufficiently supported
D5 Cultural or stylistic conjecture	Whether cultural or stylistic links are presented with appropriate evidential constraint and uncertainty	Contextual links are perceived as appropriate, constrained, and connected to visual or art-historical evidence, without overstating uncertain associations	Contextual links are perceived as weakly connected to visible evidence or art-historical context
D6 Learning inspiration	The extent to which the interpretation provides perspectives, concepts, or questions that support further looking, comparison, or interpretation	The interpretation is perceived as offering concepts, questions, or interpretive routes that support further understanding	The interpretation merely repeats obvious visual description without adding concepts, questions, or interpretive routes for further learning
D7 Bias-related perceived quality	Whether students perceive the AI-generated interpretation as avoiding obvious cultural simplification, stereotyping, or bias-related overgeneralization	Few salient bias-related problems are perceived in the interpretation	Cultural simplification, stereotyping, or overgeneralized cultural claims are perceived

Pelowski et al.'s (2016) Vienna Integrated Model further emphasizes the interaction of artwork stimuli, viewer expectations, prior knowledge, and viewing goals. In an AI-assisted setting, domain knowledge and artwork familiarity may provide reference points for checking whether fluent explanations are grounded in visible and contextual evidence. Viewing goals may also direct attention toward usefulness, plausibility, or cultural caution (Di Plinio et al., 2025). Students' ratings can therefore reflect both properties of the text and the evaluative resources they bring to the task. This framework links the evidence demands of art interpretation with the empirical analysis of rating coherence and background associations.

### 2.2 Psychological mechanisms of AI-assisted learning

Classic scaffolding accounts describe how prompts, models, and structured steps can help learners enter complex tasks (Vygotsky and Cole, 1978; Wood et al., 1976). In art learning, an AI interpretation can supply terminology, direct attention to formal features, and model connections between observations and claims. Students may then use the image and contextual sources to examine those connections. The present survey captures how useful and credible students found this form of support, while durable learning remains a question for retention, transfer, and independent-analysis measures.

Cognitive offloading can reduce the effort required to organize evidence and inference (Salomon et al., 1991; Risko and Gilbert, 2016). This support may help students begin an unfamiliar analysis, but fluent output can also create an impression of understanding before its claims have been checked (Kasneci et al., 2023). The “hallucination of learning” and students' use of epistemic safeguarding after encountering hallucinations highlight the same educational tension (Cecilio-Fernandes and Sandars, 2025; Sivenas, 2025). AI assistance is most productive when reduced initial effort is followed by active comparison of the text with visual and art-historical evidence.

### 2.3 Explicit AI-source context

Research on AI and human labels shows that source attribution can alter evaluations of creative outputs (Horton et al., 2023; Dietvorst et al., 2015; van Hees et al., 2025). Students in this study knew before rating that the interpretations had been generated by ChatGPT. The disclosed source may have shaped both their expectations of linguistic fluency and their attention to unsupported claims. The findings characterize this common use condition, while attribution effects require comparisons with human-labeled or unlabeled versions of the same texts.

Students evaluated explanatory texts about human-made artworks. This focus places language, evidence, usefulness, and cultural caution at the center of the rating task. A fluent explanation may support an overall quality impression, whereas contextual and bias-related claims may invite closer scrutiny. The disclosed-source condition thus connects expectations about AI with the different evidence demands of art interpretation.

## 3 Research questions and hypotheses

Five research questions guide the analysis. RQ1 asks whether ratings differ across the seven items. RQ2 examines whether D1–D6 are more closely associated with one another than with D7. RQ3 tests associations between ratings and art history or visual culture coursework, prior AI-art-analysis experience, AI training, and self-rated AI competence. RQ4 examines the association between artwork familiarity and the seven ratings. RQ5 describes the roles students select for ChatGPT-generated interpretation in art learning and whether the distribution of those roles differs across general AI-use frequency groups.

H1 predicts higher ratings for relatively concrete and classifiable items, particularly school or period identification and formal analysis detail, and a lower, more dispersed distribution for D7. Because D7 is positively keyed, lower values represent a greater perception of bias-related problems. H2 predicts stronger positive correlations within D1–D6 than between D7 and D1–D6. H2 treats these associations as features of the response structure without presuming seven independent latent constructs.

H3 predicts positive associations of art history coursework, prior AI-art-analysis experience, and self-rated AI competence with student ratings. H4 predicts a positive association between artwork familiarity and ratings. H5 predicts that general AI-use frequency will have a smaller conditional association with ratings after domain-specific experience, AI training, self-rated competence, and familiarity are included. H5 is evaluated from the estimated coefficient and its uncertainty; a non-significant coefficient alone is not treated as confirmation of the hypothesis.

## 4 Methods

### 4.1 Study design

This study adopted a cross-sectional online survey design. The questionnaire was distributed in February 2026 to undergraduate students in the School of Art at a comprehensive university in China. During data cleaning, responses completed in less than 3 min and records showing obvious patterned answering were removed. After cleaning, 134 valid responses remained and were used for the subsequent statistical analyses.

**Figure 1:**
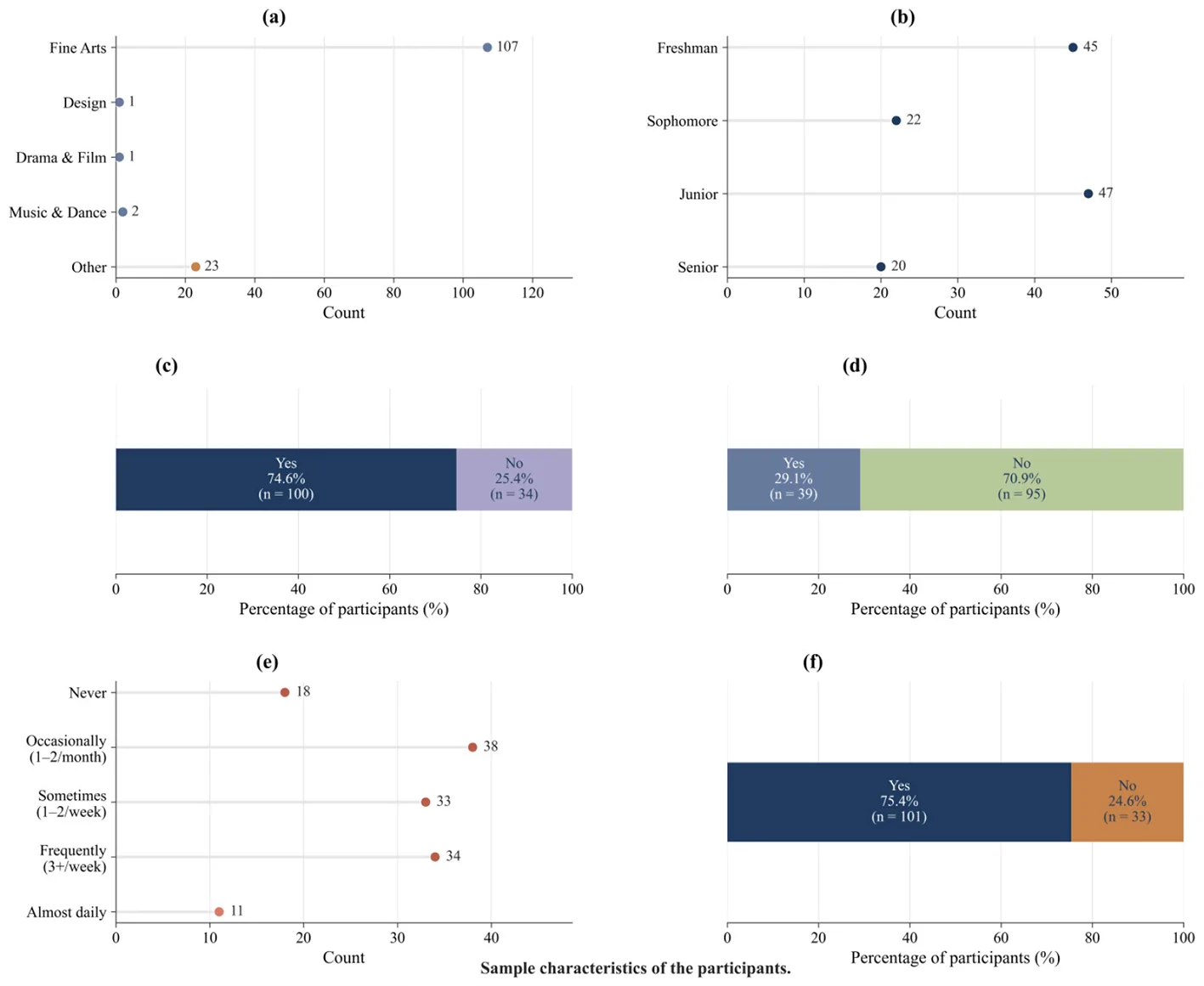
Sample characteristics of the participants. Distribution of the sample by major category, year of study, art history or visual culture coursework, AI training experience, general AI-use frequency, and prior AI-art-analysis experience. The sample is primarily composed of undergraduate fine arts students; most participants report an art history learning background but lack systematic AI training. **(a)** Major category. **(b)** Grade level. **(c)** Art history or visual culture coursework. **(d)** Systematic AI training. **(e)** AI-use frequency. **(f)** Prior AI-based artwork analysis.

As shown in Figure 1, the valid sample was concentrated in fine arts majors (*n* = 107, 79.9%). Smaller groups came from design (*n* = 1), drama and film (*n* = 1), music and dance (*n* = 2), and other categories (*n* = 23, 17.2%). The grade distribution was mainly composed of junior students (*n* = 47, 35.1%) and freshman students (*n* = 45, 33.6%), while sophomores and seniors accounted for 16.4% and 14.9%, respectively. In terms of disciplinary preparation, 100 students (74.6%) had taken courses related to art history or visual culture, whereas 39 students (29.1%) reported having received systematic training in AI tools. General AI-use frequency varied across the sample. Monthly use was reported by 28.4%, while 25.4% used AI three or more times per week and 24.6% once or twice per week. Another 13.4% never used AI, and 8.2% used it almost daily. In addition, 101 students (75.4%) reported prior experience using AI to analyze artworks. Figure 1 presents the sample distribution by major category, grade level, art history or visual culture coursework, AI training experience, general AI-use frequency, and prior AI-based artwork analysis experience.

Participants represented an undergraduate art-education context. Their familiarity with the United States artworks was expected to vary, so familiarity was measured for each stimulus and included in the subsequent analyses. Coursework and studio practice provided basic art-learning experience, while independent expert assessment remained necessary for evaluating the factual and art-historical quality of the texts.

### 4.2 Instrument design

The questionnaire comprised four sections. Section A collected major category, year of study, and coursework in art history or visual culture. It also recorded whether participants had received systematic AI tool training. Section B measured AI-use frequency, prior AI-art-analysis experience, and self-rated AI competence. Section P was the core module. Participants were sequentially shown 10 artworks from United States art together with the corresponding ChatGPT-generated analysis text. For each artwork, participants rated artwork familiarity on a 1–5 scale and evaluated the AI interpretation on seven 1–5 rating items. Section C collected perceived helpfulness through six items on 1–7 scales and role expectations through one four-option item. Two open-ended questions addressed improvement suggestions and ideal application scenarios.

**Figure 2:**
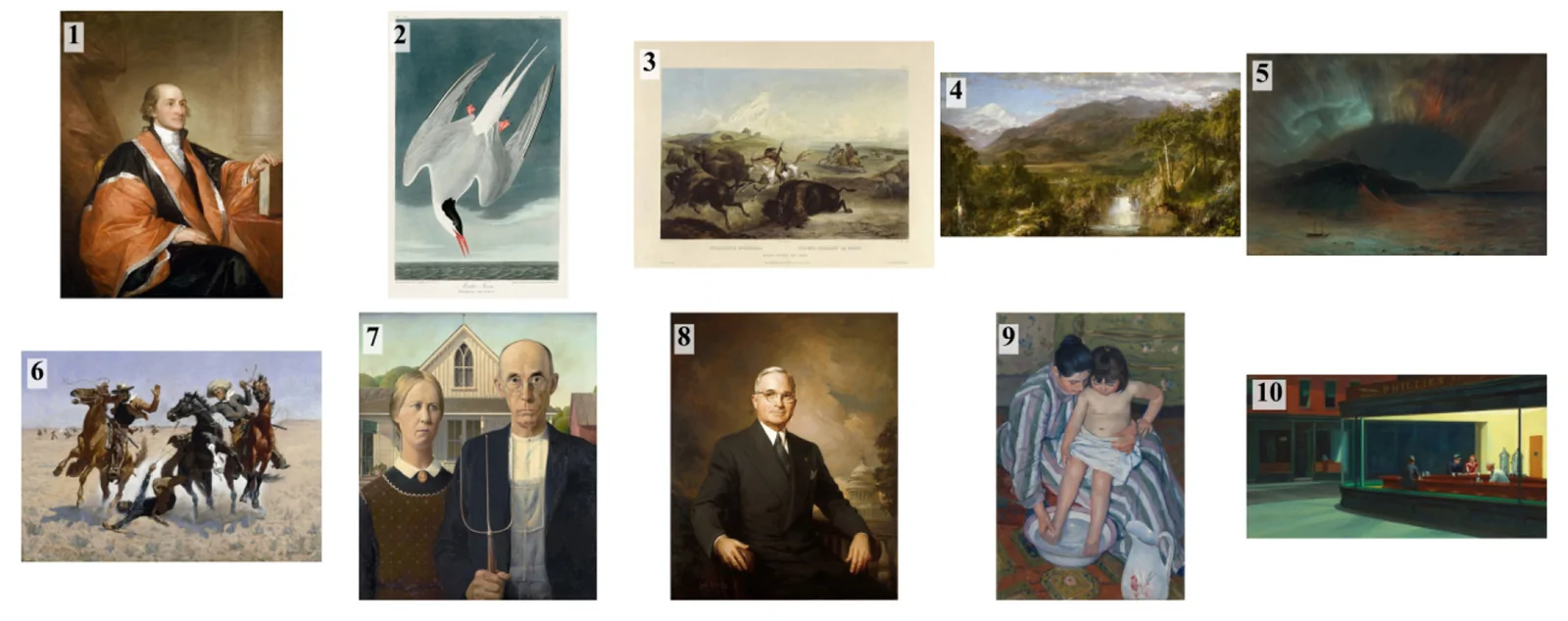
The 10 artworks used as stimuli in this study. (1) Gilbert Stuart, John Jay (1794), National Gallery of Art, Washington, DC; image obtained from Wikimedia Commons, “File:John Jay (Gilbert Stuart portrait).jpg,” identified as a public-domain work and a faithful reproduction of a public-domain two-dimensional artwork. (2) John James Audubon, Arctic Tern, Plate 250 from The Birds of America (1827–1838); image digitized by the University of Pittsburgh and obtained from Wikimedia Commons, “File:250 Arctic Tern.jpg,” public domain. (3) Karl Bodmer, Indians Hunting the Bison, Tableau 31 (1839), engraved by Charles Vogel; image provided by NONAM, Nordamerika Native Museum, and obtained from Wikimedia Commons, “File:Tableau 31 Indians hunting the bison by Karl Bodmer.jpg,” public domain. (4) Frederic Edwin Church, The Heart of the Andes (1859), The Metropolitan Museum of Art; image obtained from Wikimedia Commons, “File:Church Heart of the Andes.jpg,” identified as a faithful reproduction of a public-domain artwork. (5) Frederic Edwin Church, Aurora Borealis (1865), Smithsonian American Art Museum; image obtained from Wikimedia Commons, “File:Frederic Edwin Church - Aurora Borealis - Google Art Project.jpg,” identified as a faithful reproduction of a public-domain artwork. (6) Frederic Remington, Aiding a Comrade (1890), Museum of Fine Arts, Houston; image obtained from Wikimedia Commons, “File:Frederic Remington - Aiding a Comrade - Google Art Project.jpg,” identified as a faithful reproduction of a public-domain artwork. (7) Grant Wood, American Gothic (1930), Art Institute of Chicago; image obtained from Wikimedia Commons, “File:Grant Wood - American Gothic.jpeg,” identified on its file page as a public-domain artwork or a faithful reproduction thereof. (8) Greta Kempton, Presidential Portrait of Harry S. Truman (1947), White House Collection; image sourced from the Harry S. Truman Library and obtained from Wikimedia Commons, “File:HarryTruman.jpg,” identified on its file page as public domain in the United States. (9) Mary Cassatt, The Child's Bath (1893), Art Institute of Chicago; image obtained from Wikimedia Commons, “File:Mary Cassatt - The Child's Bath - Google Art Project.jpg,” identified as a faithful reproduction of a public-domain artwork. (10) Edward Hopper, Nighthawks (1942), Art Institute of Chicago; image obtained from Wikimedia Commons, “File:Nighthawks by Edward Hopper 1942.jpg,” identified on its file page as public domain in the United States because the copyright was not renewed. The authors prepared the composite arrangement and made only presentational modifications consisting of resizing, cropping where necessary, numbering, and layout.

The 10 stimulus artworks were selected using three criteria. First, they covered major periods and types in United States art history, from eighteenth-century Federal-era portraiture to twentieth-century modernism. Second, they represented portraiture, landscape, genre scenes, scientific illustration, and official portraiture. Third, the set spanned a familiarity gradient from Hopper's *Nighthawks* and Wood's *American Gothic* to less widely known Federal-era portraits. The selected works are shown in Figure 2.

The questionnaire translated common art-interpretation tasks into seven rating items covering classification, formal description, thematic interpretation, inferential rigor, contextual conjecture, learning inspiration, and bias-related quality. Each item used a 1–5 Likert scale, where 1 indicated strong disagreement and 5 indicated strong agreement. All items were positively keyed. D7 asked whether the interpretation avoided cultural simplification, stereotyping, and bias-related overgeneralization. Higher D7 scores indicated fewer perceived problems, whereas lower scores indicated more perceived problems. Table 2 provides the complete operational definitions and scoring direction.

### 4.3 Data collection procedure

The AI-generated interpretation texts were produced through the ChatGPT web interface in February 2026, using ChatGPT-5 as the model and Chinese as the generation language. The same prompt template was used for all 10 artworks. The prompt requested approximately 300–500 Chinese characters for each artwork. It covered style or period, formal features, subject matter or theme, inferential links, cultural or stylistic context, and potential learning inspiration. It also instructed the model to avoid definitive statements when sufficient evidence was lacking. One final output was generated for each artwork; regeneration was conducted only when the text failed to meet the required length or formatting criteria. Before inclusion in the questionnaire, the researchers did not make substantive changes to the text content and only standardized paragraph breaks and punctuation.

The questionnaire was administered through an online survey platform, with an expected completion time of 30–45 min. Each artwork page presented a high-resolution color image, the corresponding ChatGPT-generated interpretation text, and the rating scales. The 10 artworks appeared in a fixed order. Before rating, participants were informed that the interpretation texts had been generated by ChatGPT. After reading each text, participants first rated their familiarity with the artwork and then evaluated its quality across seven rating items. This sequence placed every judgment within an explicit AI-source condition.

The questionnaire was completed anonymously. Participation was voluntary and based on informed consent. No personally identifiable information, such as names, student ID numbers, or contact information, was collected. Data cleaning followed predefined criteria. Records completed in less than 3 min were removed. Records giving the same score across all rating items were treated as clearly invalid. Records showing repetitive response patterns across more than 10 consecutive items were also excluded. The cleaned valid data were used for subsequent statistical analyses.

### 4.4 Statistical analysis strategy

Rating variables were summarized with the mean (M), standard deviation (SD), and median. Cronbach's α and McDonald's ω were calculated at the artwork, rating-item, and full-scale levels. Because the same students rated the same texts in a single session, large coefficients were evaluated together with inter-item correlations and principal component analysis. In this design, very high reliability can indicate item redundancy and a common halo response as well as consistency.

The primary analyses used linear mixed-effects models for the crossed repeated-rating structure. Each participant rated multiple artworks, and each artwork was rated by multiple participants across seven items. Random intercepts were included for participants and artworks. The final mixed-effects model was estimated with restricted maximum likelihood (REML), while fixed-effect model comparisons used maximum likelihood (ML). Denominator degrees of freedom were obtained with the Satterthwaite approximation. Fixed effects were reported as coefficients, standard errors, test statistics, *p* values, and 95% Wald confidence intervals. Let *y*_*pad*_ denote the rating given by participant *p* to artwork *a* on item *d*. The main model was specified as shown in Equation 1:


$$\begin{array}{l} \begin{array}{c} y_{pad}=\beta_{0}+\overset{7}{\underset{k=2}{\sum }}\beta_{k}D_{kd}+\beta_{8}F_{pa}+\beta_{9}AH_{p}+\beta_{10}PAI_{p}\\ \;+\beta_{11}TR_{p}+\beta_{12}UF_{p}+\beta_{13}SC_{p}+u_{p}+v_{a}+\varepsilon_{pad}. \end{array} \end{array}$$ (1)

In Equation 1, *D*_*kd*_ represents dummy-coded rating-item indicators, with D1 as the reference category. *F*_*pa*_ is the familiarity rating given by participant *p* for artwork *a*. Participant-level predictors include art history or visual culture coursework (*AH*_*p*_), prior AI-art-analysis experience (*PAI*_*p*_), and systematic AI training (*TR*_*p*_). They also include general AI-use frequency (*UF*_*p*_) and self-rated AI competence (*SC*_*p*_). Random intercepts for participants and artworks accounted for repeated student responses and shared stimulus effects associated with each artwork. Their distributional assumptions are given in Equation 2:


$$\begin{array}{l} u_{p}\sim N\left(0,\sigma_{u}^{2}\right),\;v_{a}\sim N\left(0,\sigma_{v}^{2}\right),\;\varepsilon_{pad}\sim N\left(0,\sigma^{2}\right). \end{array}$$ (2)

Additional models included interaction terms between rating item and prior AI-art-analysis experience, and between rating item and artwork familiarity. The interaction model was specified as shown in Equation 3:


$$\begin{array}{l} y_{pad}=\beta_{0}+\overset{7}{\underset{k=2}{\sum }}\beta_{k}D_{kd}+\beta_{F}F_{pa}+\beta_{\text{PAI}}PAI_{p}+\beta_{\text{AH}}AH_{p}\\ \;+\beta_{\text{TR}}TR_{p}+\beta_{\text{UF}}UF_{p}+\beta_{\text{SC}}SC_{p}\\ \;+\overset{7}{\underset{k=2}{\sum }}\gamma_{k}\left(D_{kd}\times PAI_{p}\right)+\overset{7}{\underset{k=2}{\sum }}\delta_{k}\left(D_{kd}\times F_{pa}\right)\\ \;+u_{p}+v_{a}+\varepsilon_{pad}. \end{array}$$ (3)

Interaction estimates from Equation 3 were used only when the model converged and produced stable estimates. When convergence problems occurred, interpretation relied on the main-effects model in Equation 1, with interaction results reported as exploratory.

H1 was tested through the fixed effect of rating item in the mixed-effects model. If the omnibus rating-item effect reached significance, pairwise comparisons among rating items were conducted from estimated marginal means with Holm adjustment. This procedure assessed whether students' perceived-quality ratings differed across the seven rating items.

H2 was tested through correlation-structure and measurement-structure analyses. Spearman correlations were first computed among the seven rating items. The average association among D1–D6 was then compared with the average association between D7 and D1–D6 after Fisher-z transformation, as shown in Equation 4:


$$\begin{array}{l} z\left(r\right)=\frac{1}{2}\ln\left(\frac{1+r}{1-r}\right). \end{array}$$ (4)

The transformed average correlation among D1–D6 was calculated using Equation 5:


$$\begin{array}{l} {\bar{z}}_{D1-D6}=\frac{1}{15}\underset{1\leq i<j\leq 6}{\sum }z\left(\rho_{ij}\right), \end{array}$$ (5)

where ρ_*ij*_ denotes the Spearman correlation between rating items *D*_*i*_ and *D*_*j*_. The transformed average correlation between D7 and the other six rating items was calculated using Equation 6:


$$\begin{array}{l} {\bar{z}}_{D7}=\frac{1}{6}\overset{6}{\underset{i=1}{\sum }}z\left(\rho_{i7}\right). \end{array}$$ (6)

The difference between the two average associations was summarized as shown in Equation 7:


$$\begin{array}{l} \Delta z={\bar{z}}_{D1-D6}-{\bar{z}}_{D7}. \end{array}$$ (7)

Principal component analysis was used descriptively to summarize the shared response pattern across the seven items. The first component was reported because it was the only component with an eigenvalue greater than 1 (eigenvalue = 5.870) and accounted for 83.86% of the variance. Exploratory factor analysis and latent measurement modeling were outside the purpose of this analysis. For multiple correlational tests, the Benjamini–Hochberg false discovery rate procedure was applied.

Group comparisons based on Mann–Whitney *U* tests, Kruskal–Wallis *H* tests, and χ^2^ tests were used as supplementary analyses when relevant. Effect sizes for nonparametric tests were reported as rank-biserial correlation, Cliff's delta, or epsilon-squared according to test type. Cohen's *d*, when reported, was used only as a descriptive standardized mean difference. Spearman's ρ was used for bivariate associations involving ordinal variables or variables with non-normal distributions.

## 5 Results

### 5.1 Measurement structure of the seven rating items

Reliability estimates were uniformly high. At the artwork level, Cronbach's α ranged from 0.914 to 0.946; at the rating-item level, α ranged from 0.955 to 0.968. Across all 70 ratings, α was 0.991 and McDonald's ω was 0.992 (Table 3). Because the same students rated all items for the same texts in one session, these magnitudes are consistent with both response consistency and substantial item redundancy.

**Table 3:** Measurement structure of the seven rating items.

Measure	Result
Artwork-level Cronbach's α range	0.914–0.946
Rating-item-level Cronbach's α range	0.955–0.968
Full-scale Cronbach's α	0.991
Full-scale McDonald's ω	0.992
D1–D6 Spearman ρ range	0.850–0.950
D7 with D1–D6 Spearman ρ range	0.484–0.577
Mean correlation among D1–D6	0.904
Mean correlation between D7 and D1–D6	0.532
Fisher-*z* comparison	*z* = 7.31, *p* < 0.001
First component eigenvalue	5.870
First component variance explained	83.86%
First-component loadings	D1 = 0.930; D2 = 0.958; D3 = 0.970; D4 = 0.970; D5 = 0.962; D6 = 0.942; D7 = 0.626
Eigenvalues for PC1–PC7	5.870; 0.659; 0.162; 0.120; 0.088; 0.056; 0.045
Variance explained for PC1–PC7	83.86%; 9.42%; 2.32%; 1.71%; 1.25%; 0.80%; 0.64%

Spearman correlations among D1–D6 ranged from 0.850 to 0.950, with a mean of 0.904. Correlations between D7 and the other items ranged from 0.484 to 0.577, with a mean of 0.532. The Fisher-*z* comparison between these sets was significant, *z* = 7.31, p < 0.001. Responses to D1–D6 therefore moved together to a much greater extent than responses involving D7. D7 remained positively associated with the other items because all items shared a favorable scoring direction, but its bias-related focus was less integrated with the common response pattern.

Principal component analysis provided a convergent descriptive summary. The first component had an eigenvalue of 5.870 and explained 83.86% of the variance. The remaining eigenvalues were 0.659, 0.162, 0.120, 0.088, 0.056, and 0.045 for PC2–PC7, respectively. First-component loadings were high for D1–D6 (0.930–0.970) and lower for D7 (0.626). The reliability, correlation, and component results collectively identify a dominant favorable textual-quality impression across D1–D6, with D7 contributing a more distinct source of evaluative variation.

### 5.2 Differences across rating items in perceived quality

The mixed-effects model showed an overall rating-item effect, *p* < 0.001. D1, school or period identification, had the highest mean (*M* = 3.81, SD = 0.86), and the medians for D1–D6 were 4.0. D7 had the lowest mean and greatest dispersion (*M* = 3.33, SD = 1.04; media*n* = 3.0). Because D7 was positively keyed, its lower score reflects less agreement that the texts avoided bias-related problems. Holm-adjusted comparisons showed higher ratings for D1–D6 than for D7, all adjusted *p* values < 0.001 (Table 4). This distribution was consistent with H1.

**Table 4:** Rating-item-level descriptive statistics and mixed-model comparisons.

Rating item	*M*	SD	Median	*Post-hoc* comparison with D7	Holm-adjusted *p*
D1 School or period identification	3.81	0.86	4.0	D1 > D7	< 0.001
D2 Formal analysis detail	3.75	0.88	4.0	D2 > D7	< 0.001
D3 Content or theme interpretation	3.74	0.89	4.0	D3 > D7	< 0.001
D4 Inferential rigor	3.72	0.90	4.0	D4 > D7	< 0.001
D5 Cultural or stylistic conjecture	3.72	0.91	4.0	D5 > D7	< 0.001
D6 Learning inspiration	3.65	0.94	4.0	D6 > D7	< 0.001
D7 Bias-related perceived quality	3.33	1.04	3.0	Reference	—

The fitted model showed a substantial participant-level random intercept and a minimal artwork-level random intercept. The participant variance component was 0.278 (SD = 0.527), the artwork variance component was 0.0015 (SD = 0.039), and the residual variance was 0.466 (SD = 0.683). The corresponding intraclass correlations were 0.373 for participants and 0.002 for artworks, with a total ICC of 0.375. Marginal *R*^2^ was 0.097 and conditional *R*^2^ was 0.435. Repeated ratings from the same participant thus accounted for considerably more clustering than differences among the 10 artworks.

Artwork-level mean ratings varied only slightly. Among the 10 stimuli, Artwork 9, Cassatt's *The Child's Bath*, received the highest overall mean score, *M* = 3.738. Artwork 2, Audubon's bird illustration, received the lowest, *M* = 3.646. The difference between these means was 0.092, in line with the small artwork-level variance component. Most observed variation was therefore associated with participants and rating items. The high correlations within D1–D6 place their small mean differences within a broad textual-quality response, with limited separation among the six judgments.

### 5.3 Background predictors of student ratings

Prior AI-art-analysis experience was positively associated with student ratings, β = 0.34, SE = 0.10, 95% Wald CI [0.14, 0.54], *t* = 3.40, *p* < 0.001 (Table 5). Students with this experience (*n* = 101, *M* = 3.78, SD = 0.67) had higher composite ratings than students without it (*n* = 33, *M* = 3.34, SD = 0.68). The supplementary Mann–Whitney test showed the same group difference, *U* = 2319.5, *p* < 0.001. Cohen's *d* = 0.656 is reported as a descriptive standardized mean difference (Table 6).

**Table 5:** Background predictors of student ratings in the mixed-effects model.

Predictor	β	SE	95% Wald CI	*t*	*p*	Interpretation
Art history or visual culture coursework	0.23	0.10	[0.03, 0.43]	2.30	0.022	Positive association
Prior AI-art-analysis experience	0.34	0.10	[0.14, 0.54]	3.40	< 0.001	Positive association
Systematic AI training	0.08	0.09	[–0.10, 0.26]	0.89	0.374	No significant association
Self-rated AI competence	0.10	0.04	[0.02, 0.18]	2.50	0.013	Small positive association
General AI-use frequency	0.02	0.03	[–0.04, 0.08]	0.67	0.504	No significant association

**Table 6:** Group-difference tests for background variables.

Grouping variable	Test	Statistic	*p*	Effect size	Interpretation
AI art-analysis experience, yes vs. no	Mann–Whitney *U*	2319.5	< 0.001	*d* = 0.656	Descriptive standardized mean difference
Art history or visual culture coursework, taken vs. not taken	Mann–Whitney *U*	2173.5	0.015	*d* = 0.500	Descriptive standardized mean difference
General AI-use frequency, five groups	Kruskal–Wallis *H*	2.860	0.582	ε^2^ = 0.000	Supplementary comparison

Disciplinary background was also associated with ratings. Students who had taken art history or visual culture courses gave higher ratings, with β = 0.23, SE = 0.10, *t* = 2.30, *p* = 0.022 (Table 5). Students with relevant coursework (*n* = 100, *M* = 3.76, SD = 0.68) rated the interpretations higher than those without it (*n* = 34, *M* = 3.42, SD = 0.71). The supplementary Mann–Whitney test yielded U = 2173.5, *p* = 0.015, with Cohen's *d* = 0.500 (Table 6). Self-rated AI competence showed a small positive association, β = 0.10, SE = 0.04, *t* = 2.50, *p* = 0.013. Systematic AI training had a smaller, non-significant estimate, β = 0.08, SE = 0.09, *t* = 0.89, *p* = 0.374.

General AI-use frequency had a small, non-significant adjusted estimate, β = 0.02, SE = 0.03, *t* = 0.67, *p* = 0.504. The five-group comparison was also non-significant, Kruskal–Wallis H = 2.860, *p* = 0.582, ε^2^ = 0.000 (Table 6). Comparisons between low-frequency and high-frequency users across the seven items had *p* values above 0.05 and |*d*| < 0.15. The estimated association was therefore small and uncertain after adjustment, leaving H5 unconfirmed.

### 5.4 Familiarity effects

Artwork familiarity was positively associated with ratings, β = 0.11, SE = 0.02, 95% Wald CI [0.07, 0.15], *t* = 5.50, *p* < 0.001. Spearman correlations had the same direction across all items, ranging from 0.176 to 0.229, with all *p* values below 0.001 (Table 7). The largest coefficient was observed for D5 cultural or stylistic conjecture, ρ = 0.229, and the smallest for D2 formal analysis detail, ρ = 0.176. D7 was also positively associated with familiarity, ρ = 0.219, *p* < 0.001; because D7 was positively keyed, this association indicates greater agreement that the text avoided bias-related problems at higher familiarity levels.

**Table 7:** Spearman correlations between artwork familiarity and ratings on the seven rating items.

Rating item	ρ	*p*
D1 school or period identification	0.216	< 0.001
D2 formal analysis detail	0.176	< 0.001
D3 content or theme interpretation	0.204	< 0.001
D4 inferential rigor	0.226	< 0.001
D5 cultural or stylistic conjecture	0.229	< 0.001
D6 learning inspiration	0.217	< 0.001
D7 bias-related perceived quality	0.219	< 0.001

**Figure 3:**
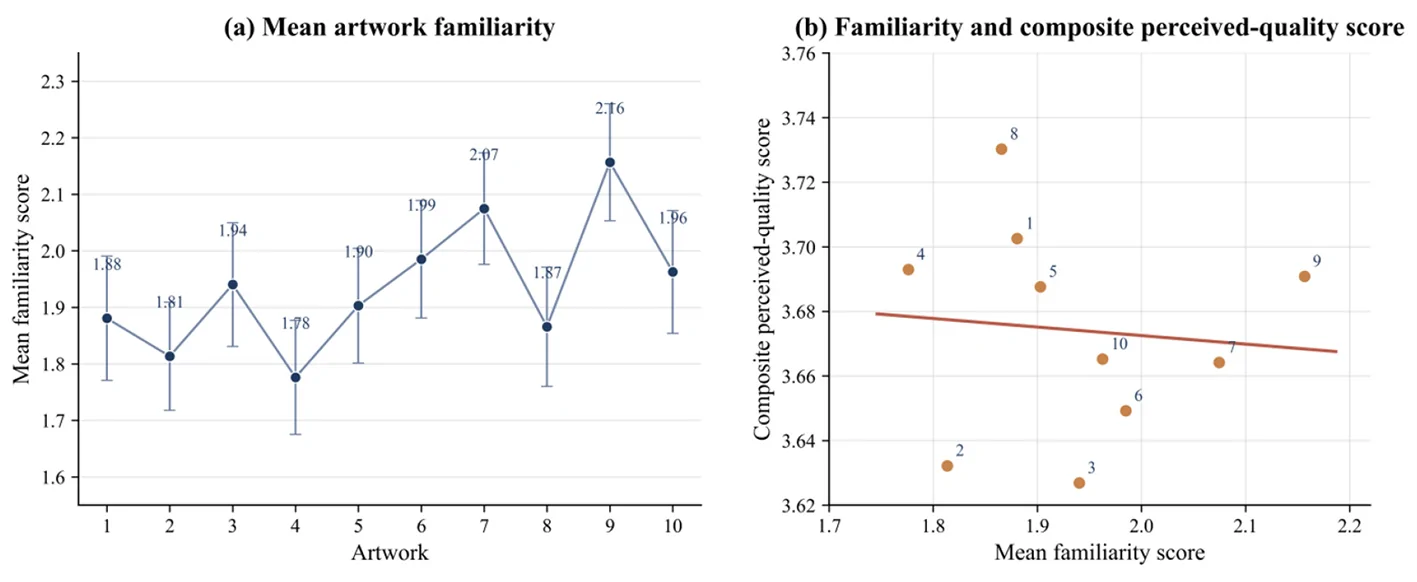
Artwork familiarity and perceived-quality ratings. **(a)** Mean familiarity for each artwork, with error bars showing variability in participant ratings. **(b)** Artwork-level mean familiarity plotted against the mean composite perceived-quality score for each of the 10 artworks. The fitted line is slightly negative at this aggregated level; the mixed-effects model and item-level Spearman correlations use all participant–artwork observations.

Participants reported low prior familiarity with the 10 artworks overall. Artwork-level familiarity means ranged from 1.78 to 2.15, indicating limited familiarity across the stimulus set. Recognizable works included Hopper's *Nighthawks* and Wood's *American Gothic*. Less commonly encountered stimuli included Federal-era portraits and Hudson River School landscapes (Figure 3a).

The positive mixed-model coefficient and item correlations were estimated from all participant–artwork observations. Figure 3b instead plots the 10 artwork-level aggregate means, for which the fitted line is slightly negative. Aggregation removes within-artwork participant covariation and leaves only 10 points, so panel b summarizes between-artwork averages at a different analytical level. At the participant–artwork level, familiarity had a consistent but modest association with ratings, with somewhat larger correlations for contextual and bias-related judgments.

### 5.5 Role expectations for AI in art learning

The six helpfulness items were rated on a 1–7 scale, and all item means were above the neutral level. Students gave the strongest endorsement to “helping identify formal features such as composition and color,” *M* = 5.29, SD = 1.31. “Helping generate appreciation-related keywords and terminology” ranked second, *M* = 5.21, SD = 1.35. “Helping understand formal meaning and value frameworks” also received a relatively high score, *M* = 5.12, SD = 1.34. “Willingness to continue using AI assistance” had the lowest mean, *M* = 5.06, SD = 1.52, and the greatest dispersion (Table 8). The perceived value of the texts was thus concentrated in formal-feature recognition, terminology, and initial explanation.

**Table 8:** Global helpfulness ratings of AI-generated interpretation support, 1–7 scale.

Item	*M*	SD
Helps identify formal features, e.g., composition, color	5.29	1.31
Helps generate appreciation keywords and terminology	5.21	1.35
Helps understand formal meaning and value frameworks	5.12	1.34
Translates cultural context into design-oriented insights	5.11	1.41
Draws attention to evidence–inference boundaries	5.07	1.38
Willing to continue using AI assistance	5.06	1.52

**Figure 4:**
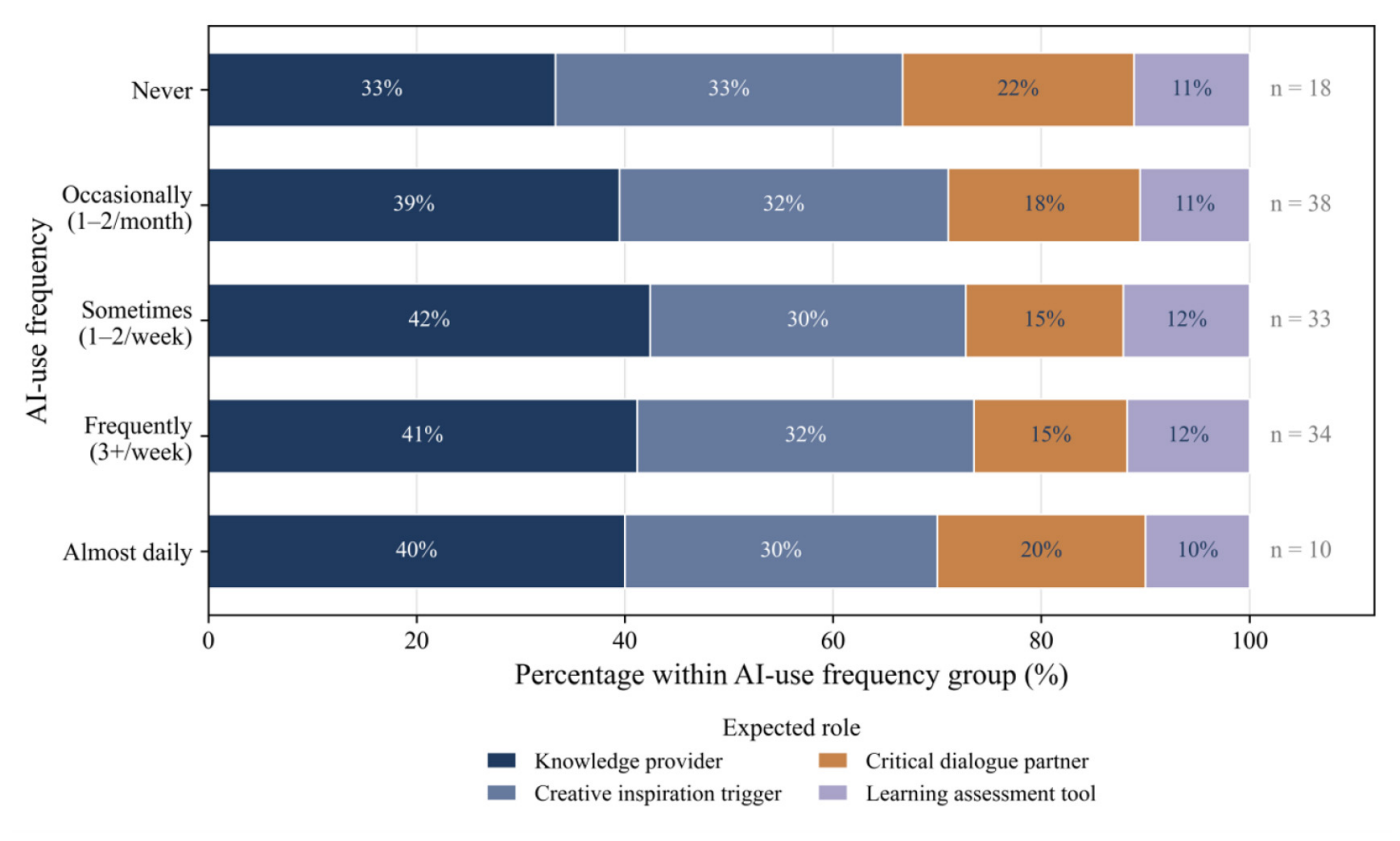
Students' role expectations for ChatGPT-generated art interpretation texts across general AI-use frequency groups.

Role expectations were concentrated in explanatory and generative functions. The largest proportion of students selected “knowledge provider” (39.6%), followed by “creative inspiration trigger” (31.3%). Together, these two roles accounted for more than 70% of the sample. Fewer students selected “critical dialogue partner” (17.2%) or “learning assessment tool” (11.2%; Table 9). Figure 4 shows a broadly similar distribution across general AI-use frequency groups. Knowledge provision and creative inspiration remained dominant, while critical dialogue and assessment were selected less frequently. The chi-square test found no significant association between general AI-use frequency and role preference, χ^2^_(12)_ = 13.42, *p* = 0.339.

**Table 9:** Distribution of students' expectations for AI roles.

Expected role	Count, *n*	Percentage, %
Knowledge provider	53	39.6
Creative inspiration trigger	42	31.3
Critical dialogue partner	23	17.2
Learning assessment tool	15	11.2

One participant did not provide a valid response to the role-expectation item; therefore, percentages for role expectations are based on *n* = 133.

Across the helpfulness and role measures, students positioned AI primarily as an aid for noticing, terminology, explanation, and idea generation. Critical-dialogue and assessment roles received less endorsement.

## 6 Discussion

Under explicit AI-source disclosure, students rated most aspects of the ChatGPT interpretations moderately positively. D1–D6 formed a closely aligned group, whereas D7 had the lowest mean, weakest integration with the other items, and widest dispersion. Because D7 was positively keyed, its distribution reflects lower and less consensual confidence that the texts avoided cultural simplification, stereotyping, and overgeneralization. Students therefore appeared to share a relatively stable impression of general textual quality while differing more in their evaluation of bias-related quality.

The high correlations among D1–D6 may reflect an overall impression formed from fluency, detail, coherence, and usefulness. These qualities are difficult to separate when students evaluate the same text in one sitting. Clear formal description can make thematic interpretation seem more plausible, while coherent reasoning can increase perceived learning value. The adjacency of the six favorable statements may also have encouraged ratings to move together. D7 requires a different kind of scrutiny. Cultural simplification and overgeneralization are less directly visible than composition or color and may depend more on contextual knowledge and cultural sensitivity. Confidence in challenging fluent prose may also vary. Its weaker correlations and greater dispersion are consistent with heterogeneous evaluative resources across students, especially when familiarity with the United States artworks was generally low.

Ratings were higher among students with prior AI-art-analysis experience, art history or visual culture coursework, greater artwork familiarity, and higher self-rated AI competence. Art-historical study and familiarity may provide schemas for recognizing plausible classifications, linking formal cues to context, and judging whether cultural claims are adequately supported. Prior AI-art-analysis experience may similarly offer reference points for typical output strengths and weaknesses, while self-rated competence may be related to confidence in evaluating generated text. General AI-use frequency showed a much weaker relationship, perhaps because routine use across unrelated tasks provides little art-specific knowledge or verification practice. The observed associations may therefore reflect evaluative familiarity and domain-specific confidence, although longitudinal or training designs are needed to examine how these capacities develop.

Students' role expectations further clarify the educational setting in which these judgments occurred. Knowledge provision and creative inspiration were selected more often than critical dialogue or learning assessment, and helpfulness ratings were highest for noticing formal features and generating terminology. This orientation may reflect the immediate accessibility of fluent explanations and the reduced effort required to begin an analysis. Under source disclosure, students may recognize fluency as an AI feature while attending more closely to hallucinated evidence or broad cultural claims (Ji et al., 2023; Kasneci et al., 2023). Art instruction can use this tension productively by asking students to locate visual support, separate observation from inference, compare alternative interpretations, and verify historical claims. Such activities position AI text as material for observation, discussion, and evidence checking while preserving the student's role in interpretation.

The findings characterize students' evaluative responses under the disclosed-source condition and clarify how perceived quality was organized across repeated ratings. Objective accuracy requires independent expert verification, while learning consequences require direct performance measures and temporal comparison. These boundaries define the population, stimulus set, and outcomes to which the interpretation applies and motivate the limitations below.

## 7 Limitations

All participants evaluated the texts under a single disclosed AI-source condition, and the sample came from one university. The results therefore represent one institutional context and one attribution setting. Familiarity was also low for several artworks from United States art traditions, which may have reduced the contextual reference points available to students. Future studies could compare matched AI, human, and unlabeled source conditions across institutions and include artworks with systematically varied cultural proximity and familiarity.

The 10 interpretation texts lacked independent expert verification, and the cross-sectional survey measured evaluative responses without direct learning outcomes. Expert review could assess factual accuracy, evidential grounding, and cultural generalization alongside student ratings. Experimental or longitudinal designs could add pre-post assessments, transfer tasks, and independent art-analysis performance to examine how AI-supported interpretation relates to learning over time. These additions would also help distinguish stable differences in domain preparation from changes associated with instruction or repeated AI use.

## 8 Conclusion

Under explicit AI-source disclosure, undergraduate art students evaluated ChatGPT-generated art interpretations moderately positively overall. D1–D6 primarily reflected a shared textual-quality impression, whereas D7 showed a more independent and divided pattern of bias-related evaluation. Domain-relevant experience and artwork familiarity were more closely associated with ratings than general AI-use frequency. In art education, AI-generated interpretations are well suited to observation, discussion, and evidence-verification activities that connect fluent claims with visible features, art-historical sources, and careful examination of cultural generalizations.

## Data Availability

The raw data supporting the conclusions of this article will be made available by the authors, without undue reservation.
